# Socioeconomic factors and other sources of variation in the prevalence of genital chlamydia infections: A systematic review and meta-analysis

**DOI:** 10.1186/s12889-015-2069-7

**Published:** 2015-07-30

**Authors:** Joanna Crichton, Matthew Hickman, Rona Campbell, Harriet Batista-Ferrer, John Macleod

**Affiliations:** School of Social and Community Medicine, University of Bristol, Canynge Hall, 39 Whatley Road, Bristol, BS8 2PS UK; School of Social and Community Medicine, University of Bristol, Oakfield House, Oakfield Grove, Bristol, BS8 2BN UK

**Keywords:** Chlamydia, Sexually transmitted infections, Young people, Socioeconomic inequalities, Systematic review

## Abstract

**Background:**

The success of chlamydia screening programmes relies on their ability to effectively target those with greatest need. Young people from disadvantaged backgrounds may be at greater need for chlamydia screening, but existing evidence on the variation of prevalence with social position is inconclusive. We carried out a systematic review to examine variation in chlamydia prevalence in populations and possible sources of this variation.

**Methods:**

Studies were eligible if they reported chlamydia prevalence derived from population-based samples that included young people aged 15–24 years from Europe, North America or Australia. Systematic searches of the following databases were undertaken from their inception to November 2014: MEDLINE, Embase, Web of Science and PsychINFO. There were no restrictions by language or publication date. Independent screening for eligibility and data extraction were carried out by two reviewers. Where possible, data were pooled in a meta-analysis using a random effects model. Heterogeneity was further investigated using meta-regression techniques.

**Results:**

Of 1248 unique titles and abstracts and 263 potentially relevant full texts, 29 studies were eligible for inclusion. There was relatively strong evidence that disadvantaged young people had an increased risk of having a chlamydia infection across multiple measures of disadvantage, including lower educational attainment (OR 1.94, 95 % CI: 1.52 to 2.47), lower occupational class (OR 1.49, 95 % CI: 1.07 to 2.08) and residence in deprived areas (OR 1.76, 95 % CI: 1.15 to 2.71) with an overall OR of 1.66 (95 % CI: 1.37 to 2.02). Socioeconomic disadvantage was associated with chlamydia infection in both men and women. There was weaker evidence that prevalence estimates also varied by gender and age.

**Conclusions:**

This review provides evidence of a consistent association between socioeconomic disadvantage and higher risk of Chlamydia infection. This association may reflect a number of factors including social variation in engagement with Chlamydia control programmes. Chlamydia screening could therefore reduce or increase health inequalities, depending on service provision and uptake by different socioeconomic groups.

**Electronic supplementary material:**

The online version of this article (doi:10.1186/s12889-015-2069-7) contains supplementary material, which is available to authorized users.

## Background

The success of chlamydia screening programmes is likely to depend on their ability to effectively reach individuals at greater risk of chlamydia [1]. In England, recent policy guidance for the National Chlamydia Screening Programmes seeks both to increase the proportion of screening participants who test positive and to ensure high levels of coverage [2]. Information on the distribution of risk of chlamydia infections in populations can be used to inform screening strategy and the impact of particular strategies can be monitored directly or explored through modeling [3–8]. Screening programmes that fail to reach those at greatest risk may exacerbate existing health inequalities [9].

Socioeconomic position has been considered as a potential source of variation in chlamydia prevalence [4, 5, 10]. Although there is substantial evidence of socioeconomic inequalities in other aspects of sexual health [11, 12], evidence is less clear as to whether this is true for chlamydia infections. A previous qualitative systematic review found inconclusive evidence that chlamydia infections vary by socioeconomic position [10].

Surveillance data on positivity among chlamydia screening service users suggests that risk of infection is higher among women than men and is higher among men aged 19 to 24 years than younger men [8, 13, 14]. However, routine health service data are unlikely to yield valid estimates of true prevalence in the general population, because those who use health services may differ systematically from those who do not, and data on non-users are usually unavailable [15–18]. Population-based studies based on representative samples are the observational method with the greatest level of external validity for estimating prevalence and the distribution of risk of infection in a population. However, population-based studies provide wide variations in prevalence estimates, sometimes even in the same population, and are often underpowered to identify any differences by population sub-group (e.g., as defined by gender or age [15–17]). Various types of participation bias, resulting from differential participation in studies of individuals with different risks of infection, may lead to variations in population-based prevalence estimates. This question has not previously been the subject of systematic study.

This review aims to identify population-based studies of chlamydia infection in women and men aged 15–24 years in higher income countries of Europe, North America and Australia. These settings were chosen as they all have Chlamydia control programmes focused on this age group involving population testing and antibiotic treatment. We further aimed to examine evidence for variation in risk of infection by factors that may have implications for design of control programmes such as gender, age and socioeconomic position and variation by study characteristics that may be indicators of potential for selection bias such as response rate, and sexual health or general health study focus.

## Methods

### Search strategy

PRISMA guidelines were followed throughout this systematic review [19], which was conducted using an *a priori* protocol. Search strategies suitable for each bibliographic database were developed using a combination of special index search terms (including medical subject headings (MeSH)), text word searches of titles and abstracts, and synonyms for genital chlamydia infection, population-based studies and prevalence. Search strategies were reviewed by a librarian with bibliographic database expertise and refined accordingly. Details on the searches are provided in an additional file (Additional file 1). We carried out searches of MEDLINE (1950 to the present), Embase (1974 to the present), Web of Science (1900 to the present) and PsychINFO (1987 to the present) from their inception to 14^th^ November 2014. An additional hand search of references cited by relevant papers and systematic reviews was carried out by one reviewer (JC). All publications identified by the searches were imported into the Endnote X7.1 reference management software [20].

### Study selection

The eligibility criteria for this review were studies that: (i) were population-based (defined as universal or random sampling of individuals from a sampling frame that closely matched the general population in a defined geographical area); (ii) included young people aged 15–24 years; (iii) were undertaken in Europe, North America or Australia; (iv) provided original data on prevalent chlamydia infection detected by laboratory diagnostic test. There were no restrictions by language or publication date. Studies based on non fee-paying public sector schools were also eligible for inclusion.

Two reviewers (JC and HB-F) independently screened titles and abstracts of 1248 candidate studies and the full texts of 263 considered to be potentially eligible for inclusion. One author (JC) carried out quality appraisal using the Critical Appraisal Skills Programme (CASP) checklist for appraising cohort studies [21], which was adapted to serve the aims and research question for this study. Due to the observational nature of the primary studies, quality appraisal was primarily undertaken to highlight potential sources of bias. No studies were excluded because of risk of bias.

### Data extraction

Information was extracted by one reviewer (JC) on: type of study, year of data collection, specimen type, diagnostic test, age group and gender of participants, response rate, number of individuals tested, and results. This was double checked by another (HB-F). Data were extracted by gender where this was reported in the original study. If studies did not report this, data were extracted for men and women combined. For socioeconomic measures, data on the most and least socially disadvantaged subgroup were extracted, taking the least disadvantaged as the reference group. Socioeconomic measures were grouped as follows: i) measures of young person’s educational opportunities or achievement (ie number of years of schooling, or academic v vocational high school); ii) measures of young person’s occupation or employment (unemployed v employed in USA and Croatia, measure of occupational class in UK); iii) neighbourhood measure of deprivation (the UK Government’s Index of Multiple Deprivation); and iv) measures of parental income, education and/or employment. Additional information on socioeconomic measures used in included studies and comparator groups for these is provided in (Additional file 2). Unreported sample sizes, response rates, standard errors and odds ratios were calculated using data reported in retrieved papers. The characteristics of the studies were presented in tables and grouped according to geographical area. Adjusted Odds Ratios (ORs) were used where available, otherwise unadjusted ORs were used.

### Statistical analyses

Heterogeneity, or the percentage of variation between studies that cannot be attributed to within-study variation, was estimated using the Q statistic and the I^2^ statistic [22]. Evidence of heterogeneity was categorised as weak, moderate or strong, based on an I^2^ greater than 20 %, 50 % and 80 %, respectively. Random effects models were used to generate summary statistics where heterogeneity was weak or moderate. Heterogeneity in prevalence estimates was investigated by stratifying meta-analyses by gender and type of socioeconomic position measure and by meta-regression. Stratifying by type of diagnostic test was also planned, but too few studies were identified that involved low sensitivity diagnostic tests for this to be possible. Heterogeneity in estimates of associations between prevalence and socioeconomic position was investigated by stratifying meta-analyses by type of socioeconomic position measure and gender. For meta-regression analysis, dummy study-level explanatory variables were created for gender, age (binary measure of age of the majority of participants: under versus over 20 years old), region (Europe versus other regions), response rate (continuous variable), topic of study (binary measure: general health or a sexual health study), sample size (continuous variable) and the timing of data collection. The latter involved a binary variable of before or after 2006, which was used as an approximate measure of whether the study was carried out before or after screening policies for chlamydia were widely introduced [23, 24]. The topic of the study was potentially important as a sexual health focused study may have a different response pattern to a general health study. It was not possible to include socioeconomic position in the meta-regression analyses due to missing or incompatible dummy variable data. The proportion of the sample with positive results was used as the dependent variable and study-level factors as the independent variables. There was insufficient power to test for interactions between factors such as gender and age.

## Results

**Fig. 1:**
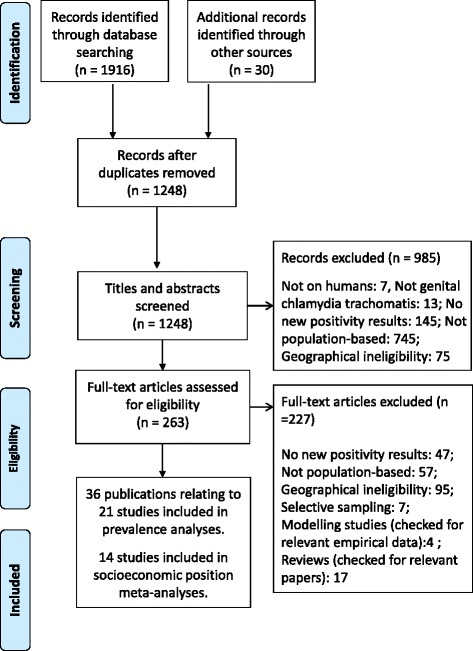
Flow diagram of records identified, included and excluded

Thirty-six papers relating to twenty-nine population-based studies of chlamydia prevalence were identified (Fig. 1). Of these, 14 studies examined variation in prevalence by socioeconomic position. Reasons for study exclusions of full texts included: lack of new positivity results (*n* = 47); not population-based (57); geographical ineligibility (95); selective sampling of high risk geographical areas (7), and; modelling studies or reviews (21).

The main characteristics of the included studies are described in Additional file 2. All studies were single country. Three (10 %) studies were carried out in the UK, nineteen (65 %) were from elsewhere in Europe (Belgium, Croatia, Denmark, Estonia, France, Germany, the Netherlands, Norway, Spain, Slovenia, Sweden), six (21 %) were from the United States of America (USA) and one was from Australia. Nine (31 %) of the studies were household surveys, ten (34 %) were postal screening surveys or trials of postal screening, four (14 %) were school-based surveys, and six (21 %) used other methods (four combined postal invitation and clinic-based data collection, one used mixed sampling methods and one was a birth cohort study). The majority of studies (24, 83 %) focused on sexual health, whereas five (17 %) studies examined health more broadly.

Twelve out of 27 (40 %) of the studies were assessed to be ‘high risk’ that the results were due to bias or chance, 16 (55 %) as ‘medium risk’ and only one study was assessed to be ‘low risk’. Risk of selection bias was a particularly common problem, with 24 studies (83 %) assessed to be at high or medium risk of having selection bias. Additional file 3 provides the risk of bias and relevance assessment for the included studies.

### Association with educational measures

**Fig. 2:**
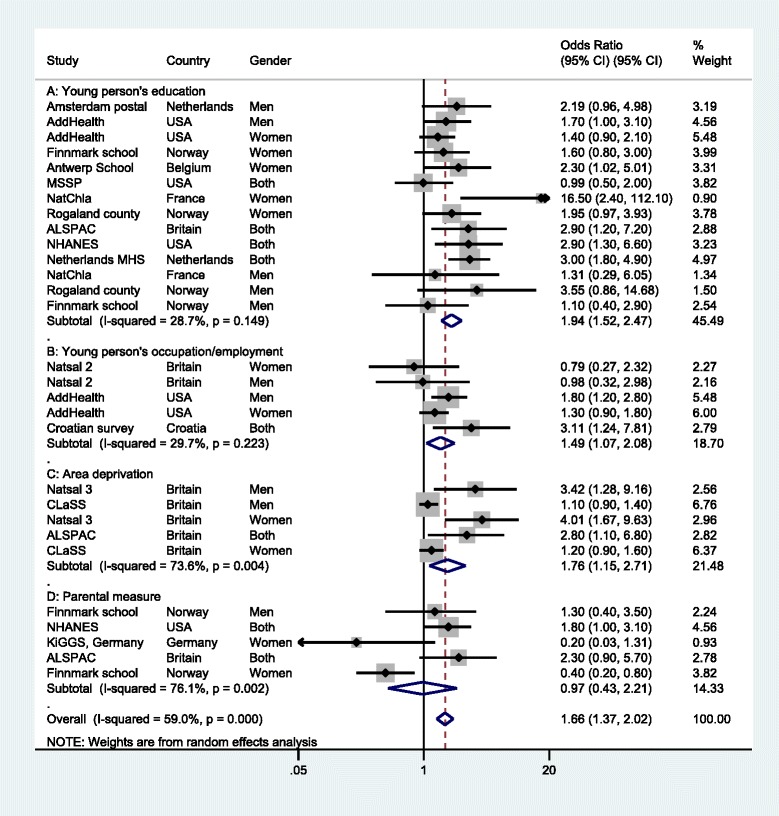
Odds ratio for chlamydia infection by socioeconomic position measures

Ten studies examined the relationship between educational opportunity or attainment of the respondent and their odds of having chlamydia infection [4, 25–33]. In most cases, educational attainment was measured by number of years in education or high school graduation. In two studies, general, academic or art schools were compared with technical and vocational schools [27, 33]. Combined results showed evidence of an association between lower educational opportunities/attainment and increased risk of chlamydia infection (Combined OR 1.94, 95 % CI: 1.52 to 2.47, I^2^ = 28.7 %, *p* = 0.149) (Fig. 2, panel a). Subgroup analysis by gender was carried out to examine whether associations varied between men and women. Combined effect estimates were similar for observations based on men, women and for studies reporting results for both sexes combined, although evidence of heterogeneity varied (Combined OR 1.89, 95 % CI: 1.24 to 2.89, I^2^ = 42.1 %, *p* = 0.141 in women; Combined OR 1.75, 95 % CI: 1.18 to 2.58, I^2^ = 0.0 %, *p* = 0.692 in men; Combined OR 2.22, 95 % CI: 1.28 to 3.87, I^2^ = 59.1 %, *p* = 0.062 in both sexes).

Three studies examined chlamydia infections by the occupation or employment of the respondent [4, 34, 35]. Results for an association with chlamydia infection were equivocal for occupational class in men or women in Britain [34] but stronger for measures of unemployment in household surveys in Croatia and the USA [4, 35]. Combining estimates gave evidence of an association between lower occupational class or unemployment and chlamydia infection (Combined OR 1.49, 95 % CI: 1.07 to 2.08, I^2^ = 29.7 %, *p* = 0.223) (Fig. 2, panel b). There were too few studies to stratify results by gender.

Three studies, all from the UK, examined associations between area deprivation and chlamydia infections [32, 36, 37]. Across both genders, there was evidence of an association between increased neighbourhood deprivation and chlamydia infection (Combined OR 1.76, 95 % CI: 1.15 to 2.71), although there was substantial evidence of heterogeneity (I^2^ = 73.6 %, *p* = 0.004) (Fig. 2, panel c). There were too few studies to stratify results by gender.

Four studies examined chlamydia prevalence using measures relating to the respondent’s parents. These included parental income, occupation or education [32, 33, 38, 39]. There was considerable evidence of heterogeneity (I^2^ = 76.1 %, *p* = 0.002) and no evidence of an association after pooling estimates (Combined OR 0.97, 95 % CI: 0.43 to 2.21). Two studies from the UK and the USA provided evidence of an association between parental socioeconomic position and chlamydia infection in both sexes, but there were equivocal results among women in Germany [38]. In rural Norway, lower maternal education was associated with decreased odds of having chlamydia in women but not in men [33].

### Prevalence and other sources of heterogeneity

Overall, there was strong evidence of heterogeneity in prevalence estimates for men (*p* < 0.001, I^2^ = 92.2 %) and for women (*p* < 0.01; I^2^ = 91.9 %). Evidence of heterogeneity among European studies remained strong after stratifying by gender, age group and region (*p* < 0.001, I^2^ = 82.4 % in women aged under 20 years; I^2^ = 83.3 %, *p* < 0.001 in women aged over 20 years; I^2^ = 85.5 %, *p* < 0.001 in men aged under 20 years and I^2^ = 80.5 %, *p* < 0.001 in men aged over 20 years) and by response rate and overall risk of bias assessment. There were too few observations among subgroups outside Europe to synthesise results.

Across studies of prevalence within and outside Europe, estimates of prevalence ranged between 0.6 % (95 % CI 0, 3.5) and 10.7 % in young women and between 0 % and 6.7 % (95 % CI 5.0, 8.8) in men (Table 1). Even within individual countries, age- and gender-specific prevalence estimates varied between studies by a factor of 2 or 3 [26, 28, 30–32, 36, 37, 39–45].

**Table 1:** Results of meta-regression models for the influence of study and subgroup characteristics on prevalence estimates (risk scale)

Study/subgroup characteristics		Number of observ-ations (studies)^a^	Unadjusted model	Mutually adjusted
			Risk difference (95 % confidence interval) *p* value	Risk difference (95 % confidence interval) *p* value
Gender	Men	24 (17)	1	1
	Women	28 (20)	0.010 (0.000, 0.020) *p* = 0.043	0.011 (0.001, 0.021) *p* = 0.031
Age	Under 20	24 (12)	1	1
	Over 20	28 (15)	0.013 (0.004, 0.023) *p* = 0.008	0.011 (0.000, 0.022) *p* = 0.048
Region	Europe	44 (17)	1	1
	Outside Europe	8 (4)	0.004 (−0.010, 0.018) *p* = 0.577	0.012 (−0.005, 0.030) *p* = 0.160
Response rate (per 10 % increase)		52 (21)	0.000 (−0.003, 0.003) *p* = 0.981	0.000 (−0.004, 0.004) *p* = 0.998
Study topic	General health	9 (4)	1	1
	Sexual health	43 (17)	0.009 (−0.004, 0.021) *p* = 0.165	0.010 (−0.006, 0.027) *p* = 0.208
Date	Before 2006	37 (17)	1	1
	After 2006	15 (5)	−0.001 (−0.013, 0.011) *p* = 0.830	0.003 (−0.008, 0.015) *p* = 0.558
Number tested (per 1000 increase in sample)		52 (21)	0.001 (−0.003, 0.004) *p* = 0.775	0.000 (−0.004, 0.004) *p* = 0.985

^a^The number of studies adds to more than 21 for gender, age and date of study because some studies reported multiple prevalence estimates for these variables

Chlamydia prevalence estimates were higher in women than men in most studies [25, 26, 28, 32–34, 36, 37, 39, 40, 46, 47]. However the confidence intervals between the sexes almost always overlapped, with the exception of under 20 year olds in four studies from Britain, Germany, the Netherlands and USA (NHANES 1999–2002) [28, 37–39].

Nine studies enabled comparison of prevalence by age [26, 29, 31, 36, 37, 39, 43, 45, 48]. Some of these studies suggested that prevalence estimates in young men may vary considerably by age, remaining low in those aged under 20 years before peaking at 20–24 years. Prevalence was greater in men aged over 20 years in five studies [26, 36, 37, 45, 48], similar in one [28] and greater in men aged 18 to 21 years in another [29]. There appeared to be less of a difference for women. In women, prevalence peaked in those aged under 20 years in three studies [28, 29, 37], was similar in another two [30, 36] and peaked in women aged over 20 years in one study [26]. The confidence intervals between age groups overlapped in most studies.

Potential sources of heterogeneity in prevalence were further explored using meta-regression analysis. Gender and age were were associated with prevalence in both univariable and mutually adjusted models (adjusted risk difference (RD) 0.011, 95 % CI: 0.001 to 0.021) for female sex and RD 0.011, 95 % CI: 0.000 to 0.022 for age over 20 years) (Table 1). There was no evidence that region, date or topic of study, response rate, or number of young people tested were associated with prevalence estimates. The adjusted R^2^ for the multivariable regression model was 21.5 % and the residual I^2^ was 79.2 %, indicating that considerable residual variation between prevalence estimates remained.

## Discussion

### Key findings

Our systematic review found strong evidence of an association between chlamydia infection and socioeconomic position in both men and women (Combined OR: 1.66, 95 % CI: 1.37 to 2.02). Pooled results were equivalent to a doubled risk of chlamydia infection for those with lower educational attainment (Combined OR: 1.94, 95 % CI: 1.52 to 2.47). Risk of infection was also greater in those with lower occupational class or unemployment and greater area deprivation. No association was found between chlamydia infection and parental or household measures of income, occupation or education.

There was considerable variability in the prevalence of chlamydia between population-based prevalence studies. This variation presumably in part reflects differences in populations and underlying prevalence between countries. However, there were also variations in estimated prevalence within populations. Evidence of heterogeneity remained strong, even after stratifying meta-analyses by gender, age and region. Prevalence estimates in young men were lower in those aged under 20 years than 20 to 24 year olds in some studies but tended to be similar in women aged under and over 20 years.

Meta-regression suggested that age and gender may contribute to the heterogeneity of prevalence estimates. Interpreted on the prevalence scale, multivariable meta-regression suggested that prevalence is on average 1.1 % (95 % CI 0.1 to 2.1 %) higher in women than men and 1.1 % (95 % CI 0.0 to 2.2 %) higher in 20 to 24 year olds compared to under 20 year olds. However, the variables included in the regression model explained only a modest amount of the between-observation variance (adjusted R^2^ = 21.5 %) and strong evidence of residual variation due to heterogeneity remained (I^2^ = 79.2 %). Other potential sources of heterogeneity include the residual influence of study characteristics such as non-response bias, sampling bias and differences in true prevalence between populations.

### Strengths and limitations

Strengths of our review include use of a pre-specified protocol, a systematic and comprehensive search strategy tailored to each bibliographic database, inclusion only of population-based studies of prevalence using an objective diagnosis of chlamydia infection, duplicate eligibility screening and data entry, and the lack of exclusions based on language or publication date. It was also possible to explore sources of heterogeneity using meta-analysis and meta-regression.

A challenge experienced in our review was the strong evidence for heterogeneity between studies even after stratifying by gender, age, and geographic region, which lead us to conclude that pooled estimates of prevalence would not be valid. Potential sources of heterogeneity between studies included study design, measurement of socioeconomic position, categorisation of reference groups and analysis of confounding. Differences in age groups and other variables between studies also limited the comparability of observations and prevented inclusion of socioeconomic position in meta-regression analyses. Ethnicity and level of urbanisation are other factors that may contribute to variation in prevalence between populations [4, 28], but were beyond the scope of the review.

The reliability of prevalence estimates in our review is limited by the risk of bias in the studies included. Overall, 95 % of the studies were assessed to be high or medium risk of important bias, particularly selection bias, in their estimates. Unfortunately, most studies included in this review had a lack of data on non-responders, which meant that it was not possible to adjust estimates for non-response using multiple imputation, inverse probability weighting or other statistical approaches to missing data [32].

Meta-regression suggested an explanation for only a small proportion of the between study variation in prevalence estimates observed in this review. In addition this approach involves the implicit assumption that true prevalence is the same in different populations. In this context, meta-regression mainly serves to identify hypotheses to be explored in future studies [49, 50]. Further, the measures of possible sources of between study variation used in meta-regression analyses in this review were relatively crude and likely to themselves be subject to measurement error. For example, response rates are only a proxy for the potential for selection bias, and whether or not data collection occurred before/after 2006 will only crudely index an influence of the introduction of Chlamydia control programmes. There were too few studies to robustly examine the influence of country where the study was carried out on variation in prevalence.

There were also limitations related to analysis of socioeconomic position in this review. Reporting bias may have led to overestimation of associations. At least one study [26] did not report the results of analyses where no association was found. Other potential sources of bias could have worked in either direction, for example potential residual confounding and adjustment of socioeconomic position for variables that may be on the causal pathway between exposure and outcome or consequences of the outcome (such as early sexual debut, number of sexual partners and symptoms of infection). Five out of thirteen observations included in the meta-analysis of educational measures were not adjusted for potential confounders, because no positive association was found in unadjusted analyses. In one study, the association between parental socioeconomic disadvantage and chlamydia was substantially attenuated and reversed in direction after adjusting for drug taking, contraceptive use and exposure to passive smoke [38]. In accordance with the study protocol, adjusted estimates were used in this review; however, both adjusted and unadjusted estimates may be subject to bias.

Some health services data and studies from some settings suggest that there are inequities in the burden of chlamydia infections between ethnic groups, with higher rates in some black ethnic groups than other black and non-black ethnic groups [4, 8, 46, 51–53]. UK population-based surveys have been inconclusive in this regard, which may reflect issues related to sample size or other methodological challenges [37, 51]. Chlamydia prevalence has also been found to vary by geographic location, including between countries, and according to urban/rural residence in some studies [28, 54] and between regions and cities in the same country in others [43, 46] Detailed consideration of these questions is beyond the scope of this paper. Our focus on variations in prevalence by age, gender and socioeconomic position in part reflected the potential importance of these demographic factors for the design of control interventions, and was because extant evidence of their association with chlamydia infection appeared inconclusive [10, 55].

### Research and policy implications

The present systematic review builds on two recent reviews [10, 55] by including more recent studies and by using meta-analysis and meta-regression to pool results and explore heterogeneity. One of these previous reviews examined inequities in prevalence by socioeconomic position and found inconclusive evidence of an association between chlamydia infection and socioeconomic position, concluding that a relationship cannot be assumed [10]. The present review provides new evidence that young people-specific and area-based measures of socioeconomic position are associated with chlamydia prevalence when pooled across studies, and finds similar inconclusive results for parental or household measures.

The second review examined risk difference in prevalence between the sexes for individual studies and found that any difference in prevalence between women and men is likely to be modest [55]. Our systematic review found weak evidence of differences in chlamydia prevalence by gender and age (under and over 20 years). These findings were from meta-regression and are best interpreted as hypotheses for testing in future research.

There are several possible mechanisms for socioeconomic inequities in chlamydia infections. These include lower engagement with Chlamydia control activities amongst disadvantaged individuals. Young people from families with lower socio-economic position may also be at greater risk of having a chlamydia infection, because individual, family, interpersonal, community and structural factors reduce the perceived benefits of safe sex, reduce consistency of condom use, reduce sexual health service provision and use, and increase other risk factors for unsafe sex, such as substance use, and mental health problems [10, 56–58]. Potential reasons for gender differences in risk for chlamydia include age differences in sexual partnerships [59], biological differences, cervical ectopy and use of hormonal contraceptives in women, and circumcision in men [55]. However, apparent differences by gender may in part be due to selection bias, which may operate in a different way in each sex [55].

Our review points to the need to monitor and address social variation in the risk of infection, in order to avoid the potential for screening programmes to exacerbate inequalities [9]. In the early years of England’s National Chlamydia Screening Programme, screening provision, coverage and positivity were all higher in socio-economically deprived areas than more affluent areas [13, 60]. However, recent population-based evidence on reported tests in the past year suggest that screening uptake is similar across all levels of neighbourhood deprivation [37]. Our findings indicate the need for greater uptake of screening in disadvantaged areas to adequately address increased risk of infection. Another potential challenge for screening programmes is ensuring sufficient uptake of tests among people aged 20 to 24 years. This age group is more difficult to reach than younger age groups who are more likely to be in full-time education. Other studies suggest that chlamydia screening uptake may be lower in men and in older age groups [13, 37]. More may need to be done to meet the need for screening in older men.

## Conclusion

This review found strong evidence of an association between chlamydia infection and measures of socioeconomic disadvantage, including respondent’s educational attainment, employment and area-based deprivation. Prevalence of chlamydia varied within the UK and across similar countries, even after stratifying by age, gender and region. There was weaker evidence that risk of infection varies by gender and age. However, there remains uncertainty about the role of study characteristics such as risk of bias and study focus (general or sexual health) in driving variation in prevalence estimates. Studies with higher response rates, larger sample sizes and analysis of data on non-responders may help to shed light on existing evidence gaps. Population-based serological studies examining the prevalence of chlamydia antibodies may also help to improve the evidence on the extent and distribution of infections [61].

## Additional files

Additional file 1:
**Search Strategy.** (PDF 49 kb) Additional file 2:
**Characteristics of included studies, estimates of prevalence and available data on socioeconomic position.** (DOCX 97 kb) Additional file 3:
**Risk of bias and relevance assessments.** (PDF 292 kb)
