# Supplementary material for: Socioeconomic factors and other sources of variation in the prevalence of genital chlamydia infections: A systematic review and meta-analysis
Source: BMC Public Health. 2015 Jul 30;15:729. doi: 10.1186/s12889-015-2069-7 (PMC4520210; doi:10.1186/s12889-015-2069-7)
Supplement: Additional file 1: — Search Strategy. (PDF 49 kb) [file 12889_2015_2069_MOESM1_ESM.pdf]

Appendix 3.1 Search Strategy

The search strategy identified publications whose title, abstract or database keywords included synonyms for i) chlamydia AND ii) sexually transmitted infection AND iii) population-based studies AND iv) prevalence.

The following search strategy was used for MEDLINE. This strategy was adapted for the other bibliographic databases using database-specific key words where appropriate:

1. Chlamydia trachomatis/
2. chlamydia.tw.
3. 1 or 2
4. urogenital.tw.
5. genital.tw.
6. sexually transmi*.tw.
7. genital diseases, male/
8. genital diseases, female/
9. sexually transmitted disease/
10. sexually transmi*.tw.
11. 4 or 5 or 6 or 7 or 8 or 9 or 10
12. 3 and 11
13. chlamydia.mp. and (genital or urogenital or sexually transmit*).tw. [mp=title, abstract, original title, name of substance word, subject heading word, keyword heading word, protocol supplementary concept word, rare disease supplementary concept word, unique identifier]
14. 12 or 13
15. limit 14 to human
16. (population adj3 stud*).tw.
17. (community adj3 stud*).tw.
18. (community adj3 sampl*).tw.
19. (population adj3 sampl*).tw.
20. population based.tw.
21. community based.tw.

22. national survey.tw.
23. probability sample survey.tw.
24. (representative adj3 sampl*).tw.
25. population estimates.tw.
26. (general adj3 population).tw.
27. population.ti.
28. 16 or 17 or 18 or 19 or 20 or 21 or 22 or 23 or 24 or 25 or 26 or 27
29. prevalen*.tw.
30. frequency.tw.
31. prevalence/
32. 29 or 30 or 31
33. 28 and 32
34. 15 and 33
