# Supplementary material for: Socioeconomic factors and other sources of variation in the prevalence of genital chlamydia infections: A systematic review and meta-analysis
Source: BMC Public Health. 2015 Jul 30;15:729. doi: 10.1186/s12889-015-2069-7 (PMC4520210; doi:10.1186/s12889-015-2069-7)
Supplement: Additional file 2: — Characteristics of included studies, estimates of prevalence and available data on socioeconomic position. (DOCX 97 kb) [file 12889_2015_2069_MOESM2_ESM.docx]

**Additional File 2: Characteristics of included studies, estimates prevalence and available data on socioeconomic position**

| **Study, country. First author^a^** | | **Type of study (sampling frame)** | **Year(s) samples taken** | **Specimen type (diagnostic test)** | **Gender and age (years) for prevalence estimates** | **Response rate** | **Number tested** | **Prevalence**  **% (95% CI)** | **Gender and age (years) for Socio-economic position (SEP) estimates** | **SEP measure** | **Comparator group** | **Data extracted** | **Variables adjusted** |
| --- | --- | --- | --- | --- | --- | --- | --- | --- | --- | --- | --- | --- | --- |
|  | **United Kingdom** | | | | | | | | | | | | |
| CLaSS, England. Macleod; Low[1, 2] | | Postal survey and screening trial (GP registers) | 2001-2002 | F: vaginal swab  M: urine  (NAATx 2) | F 16-19 | 32% ^b^ | 893 | 6.0 (4.6, 8.4) | F 16-24;  M 16-24 | Deprivation of GP (per 10% increase in IMD) | N/A | OR, aOR | Age, marital status, ethnic group, GP population ethnicity & screening uptake rate |
|  |  |  |  |  | M 16-19 | 32% ^b^ | 684 | 3.5 (2.3, 5.2) |  |  |  |  |  |
|  |  |  |  |  | F 20-24 | 32% ^b^ | 1211 | 6.2 (4.9, 8.4) |  |  |  |  |  |
|  |  |  |  |  | M 20-24 | 32% ^b^ | 762 | 6.7 (5.0, 8.8) |  |  |  |  |  |
|  | |  |  |  |  |  |  |  |  |  |  |  |  |
| National Survey of Sexual Attitudes &  Lifestyles (Natsal-2 and Natsal-3), Britain. Fenton; Sonnenberg[3, 4] | | Household survey (nationally  representative  sample, postcodes) | 1999-2001 | urine | F 18-24 | 46% ^bc^ | 379 | 3.0 (1.7, 5.0) | F 18-44;  M 18-44 | Occupational class | IV and V | OR | N/A |
|  |  |  |  | (NAAT) | M 18-24 | 46% ^bc^ | 301 | 2.7 (1.2, 5.8) |  |  |  |  |  |
|  |  |  | 2010-2012 | urine | F 16-17 | 38% ^bc^ | 171 | 2.3 (0.9, 5.8) | F 16-44;  M 16-44 | Index of Multiple Deprivation (IMD) | Most two deprived quintiles | OR, aOR | Age, number of sexual partners |
|  |  |  |  | (NAATx 2) | M 16-17 | 38% ^bc^ | 150 | 0.0 |  |  |  |  |  |
|  |  |  |  |  | F 18-19 | 38% ^bc^ | 224 | 4.7 (2.5, 8.6) |  |  |  |  |  |
|  |  |  |  |  | M 18-19 | 38% ^bc^ | 193 | 0.5 (0.1, 2.2) |  |  |  |  |  |
|  |  |  |  |  | F 20-24 | 38% ^bc^ | 597 | 2.7 (1.7, 4.2) |  |  |  |  |  |
|  |  |  |  |  | M 20-24 | 38% ^bc^ | 497 | 3.4 (2.2, 5.2) |  |  |  |  |  |
|  | |  |  |  |  |  |  |  |  |  |  |  |  |
| Avon Longitudinal Study of Parents and Children (ALSPAC), Avon, England[5] | | Birth cohort study, universal sampling in Avon area | 2008-2011 | urine  (NAATx 2) | F 17-18 | 31% | 1541 | 2.4 (1.1. 3.6) | F&M, 17-18 | Educational attainment | Did not achieve target level tests at 10-11 years | OR, aOR | Age, other SEP measures |
|  |  |  |  |  | M 17-18 | 31% |  |  |  | IMD | Most deprived (25%) | OR | N/A |
|  |  |  |  |  |  |  |  |  |  | Mother’s education | CSE/ vocational | OR, aOR | Age, other SEP measures |

| **Study, country. First author^a^** | | **Type of study (sampling frame)** | **Year(s) samples taken** | **Specimen type (diagnostic test)** | **Gender and age (years) for prevalence estimates** | **Response rate** | **Number tested** | **Prevalence**  **% (95% CI)** | **Gender and age (years) for Socio-economic position (SEP) estimates** | **SEP measure** | **Comparator group** | **Data extracted** | **Variables adjusted** |
| --- | --- | --- | --- | --- | --- | --- | --- | --- | --- | --- | --- | --- | --- |
|  | **Continental Europe** | | | | | | | | | | | | |
| [Antwerp School Study], **Belgium.** Vuylsteke[6] | | School survey. (compulsory school medical check up) | 1996-7 | Urine (NAAT x 2) | F 15-23 | 88% ^b^ | 1380 | 1.4 | F 15-23 | Type of school (general/art versus vocational) | Technical/ vocational | aOR | Symptoms, partner genital complaint, early sexual debut, number of partners, history of pregnancy |
|  | |  |  |  |  |  |  |  |  |  |  |  |  |
| [Aarhus Screening Study], Denmark. Andersen[7] | | Postal Screening RCT^d^ (County health register) | 1997-8 | vaginal swab | F 21-23 | 30% | 649 | 6.5 | No SEP data reported | N/A | N/A | N/A | N/A |
|  |  |  |  | urine (NAAT) | M 21-23 | 25% | 647 | 5.9 |  | N/A | N/A | N/A | N/A |
|  | |  |  |  |  |  |  |  |  |  |  |  |  |
| Aarhaus Cluster Randomised Home Sampling Trial, Denmark. Østergaard.[8, 9] | | Postal screening trial. (Cluster random sampling of all high schools in county) | 1997 | F: vaginal flush | F 15-19 | 45% ^c^ | 867 | 5.0 | No SEP data reported | N/A | N/A | N/A | N/A |
|  |  |  |  | M: urine  (NAATx2) | M 15-19 | 33% ^c^ | 430 | 2.6 |  | N/A | N/A | N/A | N/A |
|  | |  |  |  |  |  |  |  |  |  |  |  |  |
| [Copenhagen Human papillomavirus (HPV) study], **Denmark.** Munk[10] | | Clinic-based survey. (female population in the municipality of  Copenhagen). | 1991-1993 | Cervical swab (EIA) | F 20-24 | 68% ^b^ | 252 | 10.7 | No SEP data reported | N/A | N/A | N/A | N/A |

| **Study, country. First author^a^** | **Type of study (sampling frame)** | **Year(s) samples taken** | **Specimen type (diagnostic test)** | **Gender and age (years) for prevalence estimates** | **Response rate** | **Number tested** | **Prevalence**  **% (95% CI)** | **Gender and age (years) for Socio-economic position (SEP) estimates** | **SEP measure** | **Comparator group** | **Data extracted** | **Variables adjusted** |
| --- | --- | --- | --- | --- | --- | --- | --- | --- | --- | --- | --- | --- |
| NatChla study, **France.** Goulet[11] | Postal survey. (nationally representative sample, telephone numbers) | 2005-2006 | F: vaginal swab  M: urine (NAAT) | F 18-24 | 40% | 467 | 3.6 (1.9, 6.8) | F 18-29 | Educational attainment | Low | OR, aOR | Sexual orientation, number of partners, new/casual partner |
|  |  |  |  | M 18-24 | 36% | 322 | 2.4 (1.0, 5.7) | M 18-29 | Educational attainment | Low | raw data | N/A |
|  |  |  |  |  |  |  |  |  |  |  |  |  |
| KiGGS, **Germany.** Haar[12] | Household survey (Nationally representative, population registries) | 2003-2006 | urine (NAAT) | F 15-17 | 55% | 1136 | 2.2 (1.4, 3.5) | F 15-17 | Index of parental education,  occupation & income | Low /medium social status | OR, aOR | Drug taking, oral contraception, exposure to smoky rooms |
|  |  |  |  | M 16-17 | 61% | 789 | 0.2 (0.1, 0.7) | M: No SEP data reported | N/A | N/A | N/A | N/A |
|  |  |  |  |  |  |  |  |  |  |  |  |  |
| [Amsterdam postal screening], **Netherlands.** Valkengoed[13] | Postal screening trial and survey. (GP registers) | 1996-7 | urine (NAAT) | F 15–20 | 51%^b^ | 209 | 2.4 (0.3, 4.5) | F 15–40 | Level of education (number of years) | <15 years of education | NR: “no association” | N/A |
|  |  |  |  | M 15–20 | 33%^b^ | 149 | 0.7 (0, 2.0) | M 15–40 |  | <15 years of | aOR | Age, ethnicity, health insurance type, early sexual debut, number of partners, previous infection, symptoms |
|  |  |  |  | F 21-25 | 51%^b^ | 472 | 4.4 (2.6, 6.3) |  |  | education |  |  |
|  |  |  |  | M 21-25 | 33%^b^ | 246 | 3.3 (1.0, 5.5) |  |  |  |  |  |
|  |  |  |  |  |  |  |  |  |  |  |  |  |
| [**Netherlands** MHS screening pilot]. Van Bergen; Gotz[14, 15] | Postal survey (national probability sample of Municipal Public Health Services) | 2002-2003 | urine (NAAT) | F 15-19 | 41% ^b^ | 1657 | 2.6 (1.7, 3.4) | M&F 15-29 | Level of education (level attained) | Lower vocational / lower secondary / primary | OR, aOR | Gender, age, ethnicity, urban/rural, symptoms, number of partners, new partner |
|  |  |  |  | M 15-19 | 41% ^b^ | 916 | 1.0 (0.4, 1.5) |  |  |  |  |  |
|  |  |  |  | F 20-24 | 41% ^b^ | 1869 | 1.9 (1.2, 2.7) |  |  |  |  |  |
|  |  |  |  | M 20-24 | 41% ^b^ | 1023 | 1.3 (0.7, 1.9) |  |  |  |  |  |
| **Study, country. First author^a^** | **Type of study (sampling frame)** | **Year(s) samples taken** | **Specimen type (diagnostic test)** | **Gender and age (years) for prevalence estimates** | **Response rate** | **Number tested** | **Prevalence**  **% (95% CI)** | **Gender and age (years) for Socio-economic position (SEP) estimates** | **SEP measure** | **Comparator group** | **Data extracted** | **Variables adjusted** |
| Chlamydia Screening Implementation project (CSI­). **Netherlands.** Van den Broek[16] | Postal screening trial (population registries): Amsterdam | 2008-2011 | F: vaginal swab; M: urine (NR) | F&M 16-29 | 17.2% | 24059 | 2.6 (2.4, 2.8) | No SEP data reported | N/A | N/A | N/A | N/A |
|  | Rotterdam | 2008-2011 |  | F&M 16-29 | 15.8% | 16414 | 3.7 (3.4, 3.9) |  |  |  |  |  |
|  |  |  |  |  |  |  |  |  |  |  |  |  |
| [Finnmark school study], **Norway**. Gravningen.[17] | School survey (all high schools in 5 towns) | 2009 | urine (NAAT) | F 16-20 | 79% ^c^ | 565 | 7.3 | F 16-20 | Mother’s education | ≤ High school | OR | N/A |
|  |  |  |  | M 16-20 | 79% ^c^ | 466 | 3.9 | M 16-20 |  |  | OR | N/A |
|  |  |  |  |  |  |  |  | F 16-20 | School type |  | OR | N/A |
|  |  |  |  |  |  |  |  | M 16-20 |  |  | OR | N/A |
|  |  |  |  |  |  |  |  |  |  |  |  |  |
| [Rogaland county survey], **Norway.** Klovstad.[18] | Postal survey (simple random sample from population register) | 2006 | Urine (NAAT) | F 18-21 | 18.9%^b^ | 453 | 6.6 (4.7, 9.3) | F 18-25 | Level of education (years) | ≤14 years | OR | N/A |
|  |  |  |  | M 18-21 | 11.9%^b^ | 254 | 6.3 (3.9, 10.0) | M 18-25 |  |  | OR | N/A |
|  |  |  |  | F 22-25 | 18.9%^b^ | 477 | 5.1 (3.4, 7.4) | F 18-25 | Employed | Not employed | OR | N/A |
|  |  |  |  | M 22-25 | 11.9%^b^ | 351 | 4.3 (2.6, 6.9) | M 18-25 |  |  | OR | N/A |
|  |  |  |  |  |  |  |  |  |  |  |  |  |
| [8 country HPV study]. Barcelona, **Spain.** Franceschi.[19] | Clinic based survey (random sampling from census lists from 4 communities) | 1998-2000 | cervical cells (NAAT) | F 15-24 | 27% ^c^ | 157 | 0.6 (0, 3.5) | No SEP data reported | N/A | N/A | N/A | N/A |
|  |  |  |  |  |  |  |  |  |  |  |  |  |
| [Laviana survey]. **Spain.** Fernandez-Benitez.[20] | Census sampling (recruitment in schools, GPs, by post and by telephone) | 2010-2011 | Urine (NAAT) | F 15-19 | 46% ^b^ | 88 | 2.3 (1.2, 7.2) | No SEP data reported | N/A | N/A | N/A | N/A |
|  |  |  |  | M 15-19 | 46% ^b^ | 77 | 3.9 (2.3, 9.9) |  |  |  |  |  |
|  |  |  |  | F 20-24 | 46% ^b^ | 189 | 4.8 (3.3, 8.0) |  |  |  |  |  |
|  |  |  |  | M 20-24 | 46% ^b^ | 133 | 4.5 (2.9, 8.6) |  |  |  |  |  |
| **Study, country. First author^a^** | **Type of study (sampling frame)** | **Year(s) samples taken** | **Specimen type (diagnostic test)** | **Gender and age (years) for prevalence estimates** | **Response rate** | **Number tested** | **Prevalence**  **% (95% CI)** | **Gender and age (years) for Socio-economic position (SEP) estimates** | **SEP measure** | **Comparator group** | **Data extracted** | **Variables adjusted** |
| [Swedish women’s HPV study] Umea, **Sweden.** Jonsson.[21] | Clinic-based survey (universal sampling from health register) | 1989 | Cervical & urethral swab (culture) | F 19, 21, 23, 25 | 70% | 557 | 2.7 | No SEP data reported | N/A | N/A | N/A | N/A |
|  |  |  |  |  |  |  |  |  |  |  |  |  |
| [Nattraby Study], **Sweden**. Brannstrom.[22] | Clinic-based survey (universal sampling of rural population register) | 1990 | Cervical swab (EIA, IFL) | F 15-34 | 69% | 374 | 2.7 | No SEP data reported | N/A | N/A | N/A | N/A |
|  |  |  |  |  |  |  |  |  |  |  |  |  |
| [Vasteras school study 1991-2], **Sweden.** Svensson.[23] | School survey (all female high school students aged 16-20 in city) | 1991-1992 | urine (EIA) | F 16-20 | 77% | 751 | 2.1 | No SEP data reported | N/A | N/A | N/A | N/A |
|  |  |  |  |  |  |  |  |  |  |  |  |  |
| **Central and Eastern Europe** |  |  |  |  |  |  |  |  |  |  |  |  |
| [Croatian survey]. **Croatia.** Boziecevic[24] | Household survey (Nationally  representative, census) | 2010 | urine (NAAT) | F 18-25 | 38% | 151 | 5.3 | M&F: 18-25 | Employed | Not employed | OR | N/A |
|  |  |  |  | M 18-25 | 28% | 123 | 7.3 |  |  |  |  |  |
|  |  |  |  |  |  |  |  |  |  |  |  |  |
| National Survey of Sexual Lifestyles, Attitudes and Health in **Slovenia**. Klavs.[25] | Household survey (stratified probability sample, sampling frame not reported) | 1999-2001 | urine (NAAT) | F 18–24 | 60% ^b^ | 265 | 4.1 (2.2, 7.4) | No SEP data reported | N/A | N/A | N/A | N/A |
|  |  |  |  | M 18–24 | 51% ^b^ | 252 | 4.1 (2.2, 7.4) | No SEP data reported | N/A | N/A | N/A | N/A |

| **Study, country. First author^a^** | **Type of study (sampling frame)** | **Year(s) samples taken** | **Specimen type (diagnostic test)** | **Gender and age (years) for prevalence estimates** | **Response rate** | **Number tested** | **Prevalence**  **% (95% CI)** | **Gender and age (years) for Socio-economic position (SEP) estimates** | **SEP measure** | **Comparator group** | **Data extracted** | **Variables adjusted** |
| --- | --- | --- | --- | --- | --- | --- | --- | --- | --- | --- | --- | --- |
| [Prevalence Survey Tartu, **Estonia**], Uuskula.[26] | Postal survey (stratified random sample from population registry) | 2005-2006 | F: vaginal swab. M: urine (NAATx 2) | M&F 18-35 | 34% ^b^ | 487 ^c^ | 5.4 (3.0, 7.5) | No SEP data reported | N/A | N/A | N/A | N/A |
|  |  |  |  | F 18–35 | 34% ^b^ | NR | 6.9 (3.6, 10.3) |  |  |  |  |  |
|  |  |  |  | M 18–35 | 34% ^b^ | NR | 2.7 (0.3, 5.0) |  |  |  |  |  |
|  |  |  |  |  |  |  |  |  |  |  |  |  |
| **North America** |  |  |  |  |  |  |  |  |  |  |  |  |
| CHMS, **Canada.** Rotermann.[27] | Nationally representative household survey (Census and address register) | 2009-2011 | urine (NAAT) | F&M 14-59 | 54% | 3250 | 0.7 (0.4, 1.3) | No SEP data reported | N/A | N/A | N/A | N/A |
|  |  |  |  |  |  |  |  |  |  |  |  |  |
| National Health and Nutrition Examination Survey (NHANES), **USA**. Datta; Satterwhite; Forhan.[28, 29, 30] | Multiple household surveys (Nationally  Representative, census) | 1999–2000 | urine (NAATx 2) | F&M 14–19 | 76%^bc^ | NR | 4.3 (3.1, 6.1) | M&F 14-39 | Level of education (high school) | High school or lower | aOR | Age, gender, ethnicity, income, number of partners |
|  |  | 1999-2002 | urine (NAATx 2) | F 14–19 | 76% ^bc^ | 1649 | 4.6 (3.7, 5.8) | M&F 14-39 | Household income | Below poverty level | aOR | Age, gender, ethnicity, education, number of partners |
|  |  | 1999-2002 | urine (NAATx 2) | M 14–19 | 76% ^bc^ | 1684 | 2.3 (1.5, 3.5) |  |  |  |  |  |
|  |  | 2001–2002 | urine (NAATx 2) | F&M 14–19 | 76% ^bc^ | NR | 2.5 (2.0, 3.0) |  |  |  |  |  |
|  |  | 2003–2004 | urine (NAATx 2) | F&M 14–19 | 76% ^bc^ | NR | 2.8 (1.6, 4.9) |  |  |  |  |  |
|  |  | 2003-2004 | urine (NAATx 2) | F 14–19 | 75% ^bc^ | 793 | 3.9 (2.2, 6.9) |  |  |  |  |  |
|  |  | 2005-2006 | urine (NAATx 2) | F&M 14–19 | 76% ^bc^ | NR | 1.8 (1.1, 2.9) |  |  |  |  |  |

| **Study, country. First author^a^** | **Type of study (sampling frame)** | **Year(s) samples taken** | **Specimen type (diagnostic test)** | **Gender and age (years) for prevalence estimates** | **Response rate** | **Number tested** | **Prevalence**  **% (95% CI)** | **Gender and age (years) for Socio-economic position (SEP) estimates** | **SEP measure** |  | **Data extracted** | **Variables adjusted** |
| --- | --- | --- | --- | --- | --- | --- | --- | --- | --- | --- | --- | --- |
| NHANES continued |  | 2007–2012 | urine (NAATx 2) | F&M 14–19 | 75% ^b^ | 2724 | 2.4 (1.7, 3.1) |  |  |  |  |  |
|  |  | 2007–2012 | urine (NAATx 2) | F&M 20-24 | 75% ^b^ | 1456 | 2.9 (2.1, 3.6) |  |  |  |  |  |
|  |  | 1999–2000 | urine (NAATx 2) | F 14–25 | 76% ^bc^ | NR | 4.1 (2.4, 6.8) |  |  |  |  |  |
|  |  | 2001–2002 | urine (NAATx 2) | F 14–25 | 76% ^bc^ | NR | 2.8 (1.8, 4.5) |  |  |  |  |  |
|  |  | 2003–2004 | urine (NAATx 2) | F 14–25 | 76% ^bc^ | NR | 4.3 (2.7, 6.7) |  |  |  |  |  |
|  |  | 2005-2006 | urine (NAATx 2) | F 14–25 | 76% ^bc^ | NR | 1.8 (1.1, 2.9) |  |  |  |  |  |
|  |  | 2007–2012 | urine (NAATx 2) | F 14–24 | 75% ^b^ | NR | 4.7 (3.2, 6.1) |  |  |  |  |  |
|  |  |  |  |  |  |  |  |  |  |  |  |  |
| Add Health, **USA**. Miller; Stein[31, 32] | School-based survey (Nationally representative high schools & pupils) | 2001-2002 | urine (NAAT) | F 18-26 | 66%^b^ | 7555 | 4.7 (3.9, 5.7) | F 18-26 | Level of education (high school) | < high | OR | N/A |
|  |  |  |  | M 18-26 | 66%^b^ | 6767 | 3.7 (2.9, 4.6) | M 18-26 |  | school | OR, aOR | Ethnicity, perceived STI risk, military history, housing, recent healthcare use |
|  |  |  |  |  |  |  |  | F 18-26 | Employed | Not employed | OR | N/A |
|  |  |  |  |  |  |  |  | M 18-26 |  |  | OR | N/A |
|  |  |  |  |  |  |  |  |  |  |  |  |  |
| National Survey of Adolescent Males (NSAM), **USA**. Ku.[33] | Household survey (Nationally representative census data) | 1995 | urine (NAATx2) | M 18-19 | 62% ^c^ | 470 | 3.1 | No SEP data reported | N/A | N/A | N/A | N/A |
|  |  |  |  | M 22-26 | 40% ^c^ | 995 | 4.5 |  |  |  |  |  |

| **Study, country. First author^a^** | **Type of study (sampling frame)** | **Year(s) samples taken** | **Specimen type (diagnostic test)** | **Gender and age (years) for prevalence estimates** | **Response rate** | **Number tested** | **Prevalence**  **% (95% CI)** | **Gender and age (years) for Socio-economic position (SEP) estimates** | **SEP measure** | **Comparator group** | **Data extracted** | **Variables adjusted** |
| --- | --- | --- | --- | --- | --- | --- | --- | --- | --- | --- | --- | --- |
| Monitoring STIs Survey Program (MSSP), Baltimore, **USA**. Eggleston[34] | Household survey. (Stratified probability sampling, landline telephones) | 2006–2009 | urine (NAATx2) | F&M 15-19 | 43% ^c^ | 576 | 6.6 (3.7, 9.6) | F&M 15-35 | Level of education (high school/years behind) | <high school / 2 years behind | OR, aOR | Gender, age, ethnicity, marital status |
|  |  |  |  | F&M 20-24 |  | 460 | 5.9 (3.0, 8.9) |  |  |  |  |  |
|  |  |  |  |  |  |  |  |  |  |  |  |  |
| Baltimore STD and Behavior Survey (BSBS), **USA**. Rogers[35] | Household survey (real estate property registry) | 1997-1998 | urine (NAATx2) | F&M 18-35 | 80% | 579 | 3.0 (1.4, 4.6)^c^ | No SEP data reported | N/A | N/A | N/A | N/A |
|  |  |  |  |  |  |  |  |  |  |  |  |  |
| **Australia** |  |  |  |  |  |  |  |  |  |  |  |  |
| Melbourne survey. Hocking[36] | Postal survey (simple random sample from telephone directory) | 2003-2004 | urine (NAAT) | F 18-24 | 43% | 135 | 3.7 (1.2, 8.4) | No SEP data reported | N/A | N/A | N/A | N/A |

F: female; M: male; NR: not reported

^a^ Studies in square parentheses [] did not have a reported name in publications

^b^ Response rate or count refers to a larger sample, not specifically for the subsample of the study reported here

^c^ Calculated from figures reported in publication

**References**

1. Low N, McCarthy A, Macleod J, Salisbury C, Campbell R, Roberts TE, Horner P, Skidmore S, Sterne JA, Sanford E *et al*: **Epidemiological, social, diagnostic and economic evaluation of population screening for genital chlamydial infection**. *Health technology assessment (Winchester, England)* 2007, **11**(8):iii-iv, ix-xii, 1-165.

2. Macleod J, Salisbury C, Low N, McCarthy A, Sterne JAC, Holloway A, Patel R, Sanford E, Morcom A, Horner P *et al*: **Coverage and uptake of systematic postal screening for genital Chlamydia trachomatis and prevalence of infection in the United Kingdom general population: Cross sectional study**. *British Medical Journal* 2005, **330**(7497):940-942.

3. Fenton KA, Korovessis C, Johnson AM, McCadden A, McManus S, Wellings K, Mercer CH, Carder C, Copas AJ, Nanchahal K *et al*: **Sexual behaviour in Britain: reported sexually transmitted infections and prevalent genital Chlamydia trachomatis infection.[Erratum appears in Lancet 2002 Jan 12;359(9301):174]**. *Lancet* 2001, **358**(9296):1851-1854.

4. Sonnenberg P, Clifton S, Beddows S, Field N, Soldan K, Tanton C, Mercer CH, da Silva FC, Alexander S, Copas AJ *et al*: **Prevalence, risk factors, and uptake of interventions for sexually transmitted infections in Britain: findings from the National Surveys of Sexual Attitudes and Lifestyles (Natsal)**. *The Lancet* 2013, **382**(9907):1795-1806.

5. Crichton J, Hickman M, Campbell R, Heron J, Horner P, Macleod J: **Prevalence of chlamydia in young adulthood and association with life course socioeconomic position: birth cohort study**. *PloS one* 2014, **9**(8):e104943.

6. Vuylsteke B, Vandenbruaene M, Vandenbulcke P, Van Dyck E, Laga M: **Chlamydia trachomatis prevalence and sexual behaviour among female adolescents in Belgium**. *Sexually Transmitted Infections* 1999, **75**(3):152-155.

7. Andersen B, Olesen F, Moller JK, Ostergaard L: **Population-based strategies for outreach screening of urogenital Chlamydia trachomatis infections: a randomized, controlled trial**. *Journal of Infectious Diseases* 2002, **185**(2):252-258.

8. Ostergaard L, Andersen B, Olesen F, Moller JK: **Efficacy of home sampling for screening of Chlamydia trachomatis: randomised study**. *BMJ* 1998, **317**(7150):26-27.

9. Ostergaard L, Andersen B, Moller JK, Olesen F: **Home sampling versus conventional swab sampling for screening of Chlamydia trachomatis in women: a cluster-randomized 1-year follow-up study**. *Clinical infectious diseases : an official publication of the Infectious Diseases Society of America* 2000, **31**(4):951-957.

10. Munk C, Morre SA, Kjaer SK, Poll PA, Bock JE, Meijer CJ, van den Brule AJ: **PCR-detected Chlamydia trachomatis infections from the uterine cervix of young women from the general population: prevalence and risk determinants**. *Sex Transm Dis* 1999, **26**(6):325-328.

11. Goulet V, de Barbeyrac B, Raherison S, Prudhomme M, Semaille C, Warszawski J, Grp CSF: **Prevalence of Chlamydia trachomatis: results from the first national population-based survey in France**. *Sexually Transmitted Infections* 2010, **86**(4):263-270.

12. Haar K, Bremer V, Houareau C, Meyer T, Desai S, Thamm M, Hamouda O: **Risk factors for Chlamydia trachomatis infection in adolescents: results from a representative population-based survey in Germany, 2003-2006**. *Eurosurveillance* 2013, **18**(34):18-27.

13. van Valkengoed IG, Morre SA, van den Brule AJ, Meijer CJ, Deville W, Bouter LM, Boeke AJ: **Low diagnostic accuracy of selective screening criteria for asymptomatic Chlamydia trachomatis infections in the general population**. *Sex Transm Infect* 2000, **76**(5):375-380.

14. Gotz HM, van Bergen JE, Veldhuijzen IK, Broer J, Hoebe CJ, Steyerberg EW, Coenen AJ, de Groot F, Verhooren MJ, van Schaik DT *et al*: **A prediction rule for selective screening of Chlamydia trachomatis infection**. *Sex Transm Infect* 2005, **81**(1):24-30.

15. Van Bergen J, Gotz HM, Richardus JH, Hoebe CJPA, Broer J, Coenen AJT: **Prevalence of urogenital Chlamydia trachomatis increases significantly with level of urbanisation and suggests targeted screening approaches: Results from the first national population based study in the Netherlands**. *Sexually Transmitted Infections* 2005, **81**(1):17-23.

16. van den Broek IV, van Bergen JE, Brouwers EE, Fennema JS, Gotz HM, Hoebe CJ, Koekenbier RH, Kretzschmar M, Over EA, Schmid BV *et al*: **Effectiveness of yearly, register based screening for chlamydia in the Netherlands: controlled trial with randomised stepped wedge implementation**. *BMJ* 2012, **345**:e4316.

17. Gravningen K, Furberg AS, Simonsen GS, Wilsgaard T: **Early sexual behaviour and Chlamydia trachomatis infection - a population based cross-sectional study on gender differences among adolescents in Norway**. *BMC Infect Dis* 2012, **12**:319.

18. Klovstad H, Grjibovski A, Aavitsland P: **Population based study of genital Chlamydia trachomatis prevalence and associated factors in Norway: a cross sectional study**. *BMC infectious diseases* 2012, **12**(pp 150).

19. Franceschi S, Smith JS, Van Den Brule A, Herrero R, Arslan A, Anh PTH, Bosch FX, Hieu NT, Matos E, Posso H *et al*: **Cervical infection with Chlamydia trachomatis and Neisseria gonorrhoeae in women from ten areas in four continents: A cross-sectional study**. *Sexually Transmitted Diseases* 2007, **34**(8):563-569.

20. Fernandez-Benitez C, Mejuto-Lopez P, Otero-Guerra L, Margolles-Martins MJ, Suarez-Leiva P, Vazquez F, Chlamydial Primary Care G: **Prevalence of genital Chlamydia trachomatis infection among young men and women in Spain**. *Bmc Infectious Diseases* 2013, **13**.

21. Jonsson M, Karlsson R, Rylander E, Boden E, Edlund K, Evander M, Gustavsson A, Wadell G: **The silent suffering women - A population based study on the association between reported symptoms and past and present infections of the lower genital tract**. *Genitourinary Medicine* 1995, **71**(3):158-162.

22. Brannstrom M, Josefsson GB, Cederberg A, Liljestrand J: **PREVALENCE OF GENITAL CHLAMYDIA-TRACHOMATIS INFECTION AMONG WOMEN IN A SWEDISH PRIMARY HEALTH-CARE AREA**. *Scandinavian Journal of Infectious Diseases* 1992, **24**(1):41-46.

23. Svensson LO, Mares I, Mardh PA, Olsson SE: **SCREENING VOIDED URINE FOR CHLAMYDIA-TRACHOMATIS IN ASYMPTOMATIC ADOLESCENT FEMALES**. *Acta Obstetricia Et Gynecologica Scandinavica* 1994, **73**(1):63-66.

24. Bozicevic I, Grgic I, Zidovec-Lepej S, Cakalo JI, Belak-Kovacevic S, Stulhofer A, Begovac J: **Urine-based testing for Chlamydia trachomatis among young adults in a population-based survey in Croatia: feasibility and prevalence**. *BMC Public Health* 2011, **11**:230.

25. Klavs I, Rodrigues LC, Hayes R, Wellings K, Kese D: **Prevalence of genital Chlamydia trachomatis infection in the general population of Slovenia: Serious gaps in control**. *Sexually Transmitted Infections* 2004, **80**(2):121-123.

26. Uuskula A, Kals M, Denks K, Nurm UK, Kasesalu L, DeHovitz J, McNutt LA: **The prevalence of chlamydial infection in Estonia: A population-based survey**. *International Journal of STD and AIDS* 2008, **19**(7):455-458.

27. Rotermann M, Langlois KA, Severini A, Totten S: **Prevalence of Chlamydia trachomatis and herpes simplex virus type 2: Results from the 2009 to 2011 Canadian Health Measures Survey**. *Health Reports* 2013, **24**(4):10-15.

28. Forhan SE, Gottlieb SL, Sternberg MR, Xu FJ, Datta SD, McQuillan GM, Berman SM, Markowitz LE: **Prevalence of Sexually Transmitted Infections Among Female Adolescents Aged 14 to 19 in the United States**. *Pediatrics* 2009, **124**(6):1505-1512.

29. Satterwhite CL, Joesoef MR, Datta SD, Weinstock H: **Estimates of Chlamydia trachomatis infections among men: United States**. *Sexually Transmitted Diseases* 2008, **35**(11 Suppl):S3-7.

30. Torrone EA, Johnson RE, Tian LH, Papp JR, Datta SD, Weinstock HS: **Prevalence of Neisseria gonorrhoeae Among Persons 14 to 39 Years of Age, United States, 1999 to 2008**. *Sexually Transmitted Diseases* 2013, **40**(3):202-205.

31. Miller WC, Ford CA, Morris M, Handcock MS, Schmitz JL, Hobbs MM, Cohen MS, Harris KM, Udry JR: **Prevalence of chlamydial and gonococcal infections among young adults in the United States**. *JAMA* 2004, **291**(18):2229-2236.

32. Stein CR, Kaufman JS, Ford CA, Leone PA, Feldblum PJ, Miller WC: **Screening Young Adults for Prevalent Chlamydial Infection in Community Settings**. *Annals of Epidemiology* 2008, **18**(7):560-571.

33. Ku L, St Louis M, Farshy C, Aral S, Turner CF, Lindberg LD, Sonenstein F: **Risk behaviors, medical care, and chlamydial infection among young men in the United States**. *Am J Public Health* 2002, **92**(7):1140-1143.

34. Eggleston E, Rogers SM, Turner CF, Miller WC, Roman AM, Hobbs MM, Erbelding E, Tan S, Villarroel MA, Ganapathi L: **Chlamydia trachomatis Infection Among 15-to 35-Year-Olds in Baltimore, MD**. *Sexually Transmitted Diseases* 2011, **38**(8):743-749.

35. Rogers SM, Miller HG, Miller WC, Zenilman JM, Turner CF: **NAAT-identified and self-reported gonorrhea and chlamydial infections: different at-risk population subgroups?** *Sexually Transmitted Diseases* 2002, **29**(10):588-596.

36. Hocking JS, Willis J, Tabrizi S, Fairley CK, Garland SM, Hellard M: **A chlamydia prevalence survey of young women living in Melbourne, Victoria**. *Sexual Health* 2006, **3**(4):235-240.
