# Supplementary material for: Socioeconomic factors and other sources of variation in the prevalence of genital chlamydia infections: A systematic review and meta-analysis
Source: BMC Public Health. 2015 Jul 30;15:729. doi: 10.1186/s12889-015-2069-7 (PMC4520210; doi:10.1186/s12889-015-2069-7)
Supplement: Additional file 3: — Risk of bias and relevance assessments. (PDF 292 kb) [file 12889_2015_2069_MOESM3_ESM.pdf]

Additional File 3. Risk of bias and relevance assessments

**Study**	**1. Risk of bias in sampling / recruitment**	**2. Risk of bias in response rate**	**3. Risk of bias in outcome measurement**	**4. Appropriate adjustment of SEP for confounding?**	**5. Risk of bias after statistical methods to address missing data**	**6. Any design-specific sources of bias?**	**7. Any conflicts of interests reported?**	**8. Overall risk prevalence results could be due to bias / chance**	**9. Relevance to young people in UK?**
[Aarhus Screening Study][1]	LOW	**HIGH**	LOW	N/A	**HIGH**	NO	NO	**HIGH**	MEDIUM
ALSPAC [2]	LOW	**HIGH**	LOW	YES	MEDIUM	NO	NO	MEDIUM	HIGH
[Amsterdam Postal Screening][3]	LOW	MEDIUM for women; **HIGH** for men	LOW	F: Not adjusted; M: YES	**HIGH**	YES-LOW	NO	MEDIUM	MEDIUM
[Antwerp School Study][4]	MEDIUM	LOW	LOW	YES	MEDIUM	NO	NO	MEDIUM	MEDIUM
MSSP[5]	MEDIUM	**HIGH**	LOW	YES	MEDIUM	YES-LOW	NO	MEDIUM	LOW
BSBS[6]	LOW	LOW	LOW	N/A	MEDIUM	YES-LOW	NO	MEDIUM	LOW
ClaSS[7, 8]	LOW	**HIGH**	LOW	YES	MEDIUM	NO	NO	MEDIUM	HIGH
[Copenhagen HPV Study][9]	LOW	MEDIUM	**HIGH**	N/A	**HIGH**	NO	NO	**HIGH**	MEDIUM

[Croatian survey][10]	LOW	**HIGH**	LOW	N/A	**HIGH**	YES-MEDIUM	NO	**HIGH**	MEDIUM
[Finnmark school study][11]	MEDIUM	LOW	LOW	F: YES; M: Not adjusted	**HIGH**	YES-LOW	NO	MEDIUM	LOW
[NatChla][12]	LOW	**HIGH**	LOW	F: YES; M: Not adjusted	MEDIUM	YES-LOW	NO	MEDIUM	MEDIUM
[Netherlands MHS screening pilot][13, 14]	LOW	**HIGH**	LOW	YES	MEDIUM	NO	NO	MEDIUM	MEDIUM
NSSLAH Slovenia[15]	LOW	MEDIUM	LOW	N/A	MEDIUM	NO	NO	MEDIUM	MEDIUM
[Eight country HPV study][16]	**HIGH**	**HIGH**	LOW	N/A	**HIGH**	NO	NO	**HIGH**	MEDIUM
[Swedish women's HPV study][17]	LOW	MEDIUM	**HIGH**	N/A	**HIGH**	NO	NO	**HIGH**	LOW
[Prevalence Survey Estonia][18]	LOW	**HIGH**	LOW	N/A	MEDIUM	NO	NO	**HIGH**	LOW
Natsal-2[19] & Natsal-3[20]	LOW	**HIGH**	LOW	Natsal-2: Not adjusted; Natsal-3: YES	MEDIUM	NO	NO	MEDIUM	HIGH
NHANES[21, 22]	LOW	LOW	LOW	YES	MEDIUM	NO	NO	LOW	MEDIUM
Add Health[23, 24]	MEDIUM	MEDIUM	LOW	F: Not adjusted M: YES	MEDIUM	NO	NO	MEDIUM	MEDIUM
[Nattraby Study][25]	LOW	MEDIUM	**HIGH**	N/A	**HIGH**	NO	NO	**HIGH**	LOW
Aarhaus Cluster Randomised Home Sampling Trial[26, 27]	MEDIUM	**HIGH**	LOW	N/A	**HIGH**	NO	NO	**HIGH**	MEDIUM

[Vasteras school study][28]	MEDIUM	LOW	**HIGH**	N/A	**HIGH**	YES	NO	**HIGH**	MEDIUM
NSAM[29]	LOW	MEDIUM: 18-19 year olds; **HIGH:** 22-26 year olds	LOW	N/A	UNCLEAR	NO	NO	MEDIUM	MEDIUM
[Chlamydia Prevalence in Laviana][30]	**HIGH**	**HIGH**	LOW	N/A	**HIGH**	NO	YES	**HIGH**	MEDIUM
KiGGS (German Health Interview and Examination Survey for Children and Adolescents)[31]	LOW	MEDIUM	LOW	YES	MEDIUM	NO	NO	MEDIUM	MEDIUM
[Rogaland county, Norway][32]	**HIGH**	**HIGH**	LOW	Not adjusted.	**HIGH**	NO	NO	**HIGH**	MEDIUM
Canadian Health Measures Survey (CHMS)[33]	LOW	MEDIUM	LOW	N/A	MEDIUM	NO	UNCLEAR	MEDIUM	LOW
CSI[34]	MEDIUM	**HIGH**	LOW	N/A	MEDIUM	NO	NO	**HIGH**	MEDIUM
Melbourne survey[35]	MEDIUM	**HIGH**	LOW	N/A	MEDIUM	NO	NO	MEDIUM	MEDIUM

N/A: not applicable.
Aspects of studies assessed as involving high risk of bias are highlighted in bold.
Note: Question 4 examined the appropriateness of analyses of associations between chlamydia and socioeconomic position for the purposes of this review. Studies that did not report adjusted odds ratios were classified as not adjusting appropriately, since adjusted odds ratios were required for meta-analysis in this review.

1. Andersen B, Olesen F, Moller JK, Ostergaard L: **Population-based strategies for outreach screening of urogenital Chlamydia trachomatis infections: a randomized, controlled trial**. *Journal of Infectious Diseases* 2002, **185**(2):252-258.
2. Crichton J, Hickman M, Campbell R, Heron J, Horner P, Macleod J: **Prevalence of chlamydia in young adulthood and association with life course socioeconomic position: birth cohort study**. *PloS one* 2014, **9**(8):e104943.
3. van Valkengoed IG, Morre SA, van den Brule AJ, Meijer CJ, Deville W, Bouter LM, Boeke AJ: **Low diagnostic accuracy of selective screening criteria for asymptomatic Chlamydia trachomatis infections in the general population**. *Sex Transm Infect* 2000, **76**(5):375-380.
4. Vuylsteke B, Vandenbruaene M, Vandenbulcke P, Van Dyck E, Laga M: **Chlamydia trachomatis prevalence and sexual behaviour among female adolescents in Belgium**. *Sexually Transmitted Infections* 1999, **75**(3):152-155.
5. Eggleston E, Rogers SM, Turner CF, Miller WC, Roman AM, Hobbs MM, Erbelding E, Tan S, Villarroel MA, Ganapathi L: **Chlamydia trachomatis Infection Among 15-to 35-Year-Olds in Baltimore, MD**. *Sexually Transmitted Diseases* 2011, **38**(8):743-749.
6. Rogers SM, Miller HG, Miller WC, Zenilman JM, Turner CF: **NAAT-identified and self-reported gonorrhea and chlamydial infections: different at-risk population subgroups?** *Sexually Transmitted Diseases* 2002, **29**(10):588-596.
7. Low N, McCarthy A, Macleod J, Salisbury C, Campbell R, Roberts TE, Horner P, Skidmore S, Sterne JA, Sanford E *et al*: **Epidemiological, social, diagnostic and economic evaluation of population screening for genital chlamydial infection**. *Health technology assessment (Winchester, England)* 2007, **11**(8):iii-iv, ix-xii, 1-165.
8. Macleod J, Salisbury C, Low N, McCarthy A, Sterne JAC, Holloway A, Patel R, Sanford E, Morcom A, Horner P *et al*: **Coverage and uptake of systematic postal screening for genital Chlamydia trachomatis and prevalence of infection in the United Kingdom general population: Cross sectional study**. *British Medical Journal* 2005, **330**(7497):940-942.
9. Munk C, Morre SA, Kjaer SK, Poll PA, Bock JE, Meijer CJ, van den Brule AJ: **PCR-detected Chlamydia trachomatis infections from the uterine cervix of young women from the general population: prevalence and risk determinants**. *Sex Transm Dis* 1999, **26**(6):325-328.
10. Bozicevic I, Grgic I, Zidovec-Lepej S, Cakalo JI, Belak-Kovacevic S, Stulhofer A, Begovac J: **Urine-based testing for Chlamydia trachomatis among young adults in a population-based survey in Croatia: feasibility and prevalence**. *BMC Public Health* 2011, **11**:230.
11. Gravningen K, Furberg AS, Simonsen GS, Wilsgaard T: **Early sexual behaviour and Chlamydia trachomatis infection - a population based cross-sectional study on gender differences among adolescents in Norway**. *BMC Infect Dis* 2012, **12**:319.

12. Goulet V, de Barbeyrac B, Raherison S, Prudhomme M, Semaille C, Warszawski J, Grp CSF: **Prevalence of Chlamydia trachomatis: results from the first national population-based survey in France**. *Sexually Transmitted Infections* 2010, **86**(4):263-270.
13. Van Bergen J, Gotz HM, Richardus JH, Hoebe CJPA, Broer J, Coenen AJT: **Prevalence of urogenital Chlamydia trachomatis increases significantly with level of urbanisation and suggests targeted screening approaches: Results from the first national population based study in the Netherlands**. *Sexually Transmitted Infections* 2005, **81**(1):17-23.
14. Gotz HM, van Bergen JE, Veldhuijzen IK, Broer J, Hoebe CJ, Steyerberg EW, Coenen AJ, de Groot F, Verhooren MJ, van Schaik DT *et al*: **A prediction rule for selective screening of Chlamydia trachomatis infection**. *Sex Transm Infect* 2005, **81**(1):24-30.
15. Klavs I, Rodrigues LC, Hayes R, Wellings K, Kese D: **Prevalence of genital Chlamydia trachomatis infection in the general population of Slovenia: Serious gaps in control**. *Sexually Transmitted Infections* 2004, **80**(2):121-123.
16. Franceschi S, Smith JS, Van Den Brule A, Herrero R, Arslan A, Anh PTH, Bosch FX, Hieu NT, Matos E, Posso H *et al*: **Cervical infection with Chlamydia trachomatis and Neisseria gonorrhoeae in women from ten areas in four continents: A cross-sectional study**. *Sexually Transmitted Diseases* 2007, **34**(8):563-569.
17. Jonsson M, Karlsson R, Rylander E, Boden E, Edlund K, Evander M, Gustavsson A, Wadell G: **The silent suffering women - A population based study on the association between reported symptoms and past and present infections of the lower genital tract**. *Genitourinary Medicine* 1995, **71**(3):158-162.
18. Uuskula A, Kals M, Denks K, Nurm UK, Kasesalu L, DeHovitz J, McNutt LA: **The prevalence of chlamydial infection in Estonia: A population-based survey**. *International Journal of STD and AIDS* 2008, **19**(7):455-458.
19. Fenton KA, Korovessis C, Johnson AM, McCadden A, McManus S, Wellings K, Mercer CH, Carder C, Copas AJ, Nanchahal K *et al*: **Sexual behaviour in Britain: reported sexually transmitted infections and prevalent genital Chlamydia trachomatis infection.[Erratum appears in Lancet 2002 Jan 12;359(9301):174]**. *Lancet* 2001, **358**(9296):1851-1854.
20. Sonnenberg P, Clifton S, Beddows S, Field N, Soldan K, Tanton C, Mercer CH, da Silva FC, Alexander S, Copas AJ *et al*: **Prevalence, risk factors, and uptake of interventions for sexually transmitted infections in Britain: findings from the National Surveys of Sexual Attitudes and Lifestyles (Natsal)**. *The Lancet* 2013, **382**(9907):1795-1806.
21. Datta SD, Sternberg M, Johnson RE, Berman S, Papp JR, McQuillan G, Weinstock H: **Gonorrhea and Chlamydia in the United States among Persons 14 to 39 Years of Age, 1999 to 2002**. *Annals of Internal Medicine* 2007, **147**(2):89-96.
22. Forhan SE, Gottlieb SL, Sternberg MR, Xu FJ, Datta SD, McQuillan GM, Berman SM, Markowitz LE: **Prevalence of Sexually Transmitted Infections Among Female Adolescents Aged 14 to 19 in the United States**. *Pediatrics* 2009, **124**(6):1505-1512.

23. Stein CR, Kaufman JS, Ford CA, Leone PA, Feldblum PJ, Miller WC: **Screening Young Adults for Prevalent Chlamydial Infection in Community Settings**. *Annals of Epidemiology* 2008, **18**(7):560-571.
24. Miller WC, Ford CA, Morris M, Handcock MS, Schmitz JL, Hobbs MM, Cohen MS, Harris KM, Udry JR: **Prevalence of chlamydial and gonococcal infections among young adults in the United States**. *JAMA* 2004, **291**(18):2229-2236.
25. Brannstrom M, Josefsson GB, Cederberg A, Liljestrand J: **PREVALENCE OF GENITAL CHLAMYDIA-TRACHOMATIS INFECTION AMONG WOMEN IN A SWEDISH PRIMARY HEALTH-CARE AREA**. *Scandinavian Journal of Infectious Diseases* 1992, **24**(1):41-46.
26. Ostergaard L, Andersen B, Olesen F, Moller JK: **Efficacy of home sampling for screening of Chlamydia trachomatis: randomised study**. *BMJ* 1998, **317**(7150):26-27.
27. Ostergaard L, Andersen B, Moller JK, Olesen F: **Home sampling versus conventional swab sampling for screening of Chlamydia trachomatis in women: a cluster-randomized 1-year follow-up study**. *Clinical infectious diseases : an official publication of the Infectious Diseases Society of America* 2000, **31**(4):951-957.
28. Svensson LO, Mares I, Mardh PA, Olsson SE: **SCREENING VOIDED URINE FOR CHLAMYDIA-TRACHOMATIS IN ASYMPTOMATIC ADOLESCENT FEMALES**. *Acta Obstetricia Et Gynecologica Scandinavica* 1994, **73**(1):63-66.
29. Ku L, St Louis M, Farshy C, Aral S, Turner CF, Lindberg LD, Sonenstein F: **Risk behaviors, medical care, and chlamydial infection among young men in the United States**. *Am J Public Health* 2002, **92**(7):1140-1143.
30. Fernandez-Benitez C, Mejuto-Lopez P, Otero-Guerra L, Margolles-Martins MJ, Suarez-Leiva P, Vazquez F, Chlamydial Primary Care G: **Prevalence of genital Chlamydia trachomatis infection among young men and women in Spain**. *Bmc Infectious Diseases* 2013, **13**.
31. Haar K, Bremer V, Houareau C, Meyer T, Desai S, Thamm M, Hamouda O: **Risk factors for Chlamydia trachomatis infection in adolescents: results from a representative population-based survey in Germany, 2003-2006**. *Eurosurveillance* 2013, **18**(34):18-27.
32. Klovstad H, Grjibovski A, Aavitsland P: **Population based study of genital Chlamydia trachomatis prevalence and associated factors in Norway: a cross sectional study**. *BMC infectious diseases* 2012, **12**(pp 150).
33. Rotermann M, Langlois KA, Severini A, Totten S: **Prevalence of Chlamydia trachomatis and herpes simplex virus type 2: Results from the 2009 to 2011 Canadian Health Measures Survey**. *Health Reports* 2013, **24**(4):10-15.
34. van den Broek IV, van Bergen JE, Brouwers EE, Fennema JS, Gotz HM, Hoebe CJ, Koekenbier RH, Kretzschmar M, Over EA, Schmid BV *et al*: **Effectiveness of yearly, register based screening for chlamydia in the Netherlands: controlled trial with randomised stepped wedge implementation**. *BMJ* 2012, **345**:e4316.

35. Hocking JS, Willis J, Tabrizi S, Fairley CK, Garland SM, Hellard M: **A chlamydia prevalence survey of young women living in Melbourne, Victoria**. *Sexual Health* 2006, **3**(4):235-240.
